# Preoperative weight loss is associated with poorer prognosis in operable esophageal cancer patients: A single-center retrospective analysis of a large cohort of Chinese patients

**DOI:** 10.7150/jca.40344

**Published:** 2020-02-03

**Authors:** Shuishen Zhang, Yonghuang Tan, Xiaoli Cai, Kongjia Luo, Zhongkai Wu, Jianjun Lu

**Affiliations:** 1 Department of Thoracic Surgery, the First Affiliated Hospital, Sun Yat-Sen University, Guangzhou, People's Republic of China; 2 Department of Medical Ultrasonics, First Affiliated Hospital of Jinan University, Guangzhou, People's Republic of China; 3 Department of Thoracic Oncology, Sun Yat-Sen University Cancer Center, Guangzhou, People's Republic of China; 4 Department of Cardiac Surgery, First Affiliated Hospital of Sun Yat-Sen University; Key Laboratory on Assisted Circulation, Ministry of Health, Guangzhou, People's Republic of China

**Keywords:** esophageal cancer, preoperative weight loss, disease-free survival, overall survival, prognosis factor

## Abstract

**Background**: Preoperative weight loss has been shown to be a prognostic factor for many cancers. However, whether preoperative weight loss has clinical significance in patients with esophageal cancer is still controversial.

**Methods**: A total of 2,174 Chinese patients underwent radical resection of esophageal cancer from 2000 to 2008 were included in our study. Patients were divided into two group: no weight loss (-) and weight loss (+), according to whether they had weight loss compared with their usual weight at diagnosis. The influence of preoperative weight loss on disease-free survival (DFS) and overall survival (OS) was estimated using the Kaplan-Meier method and Cox proportional hazard models.

**Results**: weight loss (+) was significantly associated with age (*P*=0.001), alcoholism (*P*<0.001), tumor location (*P*=0.003), pT category (*P*=0.003), pN category (*P*=0.001). Patients of group weight loss (+) had significantly poorer DFS (Mean: 63.3 months (m) vs 76.8 m, *P*<0.001) and OS (67.4 m vs 83.3 m, *P*<0.001) than the no weight loss (-) group. In the final multivariate survival analysis with adjustment for covariates, we found that the weight loss (+) group had a 19% higher risk of death (HR=1.19, 95%CI: 1.07-1.33, *P*=0.002) and had a 13% higher risk of disease progression (HR=1.13, 95%CI: 1.01-1.25, *P*=0.027), respectively, than the no weight loss (-) group. Subgroup analysis indicated that the association with preoperative weight loss and better DFS or OS was observed in patients with esophageal squamous cell carcinoma (ESCC) and early pathological stage (I-II).

**Conclusion**: Preoperative weight loss is associated with shorter OS and DFS, which means poor postoperative prognosis in esophageal cancer patients.

## Background

As a common gastrointestinal malignancy in China, esophageal cancer has become one of the leading causes of death. According to the latest epidemiological statistics, there were 477,900 cases and 375,000 deaths of esophageal cancer in China in 2015^1^. Although the treatment of esophageal cancer is becoming more and more comprehensive, the latest clinical trials show that the five-year survival rate for patients who have received the comprehensive treatment is about 47% 2. There are many ways to predict the prognosis of patients undergoing surgery, such as the Nutritional Risk Index (NRI), Maastricht Index (MI), Subjective Global Assessment (SGA), and Mini Nutritional Assessment (MNA) 3, but a straightforward prognostic factor for esophageal cancer may have higher clinical significance in improving its prognosis.

Weight loss is a comment symptom in approximately 57% to 85% of esophageal cancer patients will experience before surgery 4,5. The main causes of weight loss include dysphagia, decreased oral intake, systemic inflammation, and mental factors 4-6. Weight loss has been shown to be a prognostic factor in colon 7, stomach 8, cervical 9 and lung cancers 10. As to esophageal cancer, some studies have indicated that preoperative weight loss is a risk factor for prognosis. Hynes' and Van' studys showed that preoperative weight loss of >10% was a potential risk factor for the prognosis of patients undergoing radical esophagectomy. And in Yu's cohort of patients receiving adjuvant chemotherapy after radical esophagectomy indicated that the greater the weight loss, the worse the prognosis 11-13. On the contrary, other studies have suggested that weight loss is not a reliable independent prognostic factor 14. Hence, the correlation between preoperative weight loss and prognosis of esophageal cancer is controversial.

The purpose of this study was to further investigate the relationship between preoperative weight loss and prognosis among patients who received surgery for esophageal carcinoma in a large cohort of Chinese patients, thus providing more evidence to determine whether preoperative weight loss can be used as a prognostic factor for patients with esophageal cancer.

## Materials and Methods

### Patients

We identified consecutive patients with esophageal cancer who underwent radical surgery for esophageal carcinoma at Sun Yat-sen University Cancer Center between December 2000 and December 2008^15^. Patients who had received neoadjuvant or adjuvant therapy, had a history of other cancer, lacked weight loss date were excluded. Finally 2,174 patients were included based on the criteria. Patient characteristics including, weight loss were collected from retrospective medical record review using a standardized data collection form.

### Surgery

The most common surgical approaches included the left transthoracic procedure, the Ivor-Lewis approach and the cervicothoraco abdominal procedures. Lymph node dissection including standard or extended dissection of thoracic and abdominal lymph nodes was performed in patients with no evidence of metastatic disease that included cervical or coeliac lymph node metastases 16. Pathological stage was determined according to the 7th edition of the AJCC staging system. The study was approved by the Ethics Committee of Sun Yat-sen University Cancer Center. All patients provided written informed consents according to the ethical approval.

### Weight loss

Patients were asked whether they had weight loss compared with their usual weight when their weight was measured at diagnosis. Patients were divided into two group: no weight loss (-) and weight loss (+).

### Follow-up

All patients received standardized follow-up at 3-month intervals for the first 2 years after surgery, at 6-month intervals in the third year and yearly thereafter. Follow-up time was calculated from the date of surgery to the event or the date of the last contact. Follow-up continued until June 2012. The primary endpoint was overall survival (OS), which was calculated from the time of surgery to the time of death from any causes. The second endpoint was disease-free survival (DFS), which was calculated from the time of surgery to the first recurrence of index cancer or to all-cause death.

### Statistical analysis

Statistical analysis was performed using SPSS 16.0 for Windows software system (SPSS Inc, Chicago, IL). Weight loss was analyzed as a categorical variable after grouping by no weight loss (-) and weight loss (+). The association between weight loss and clinicopathologic parameters was analyzed by χ^2^-test or Fisher's exact test. Survival curves were depicted by the Kaplan-Meier method and analyzed by log-rank test. Multivariate analysis was performed using Cox's proportional hazards regression model with a forward stepwise procedure (the entry and removal probabilities were 0.05 and 0.10, respectively). We tested the proportional hazards assumption by Shoenfeld residuals test and found the test is not statistically significant for each of the covariates as well as the global test. Therefore, we can assume the proportional hazards. A significant difference was declared if the *P* value from a two-tailed test was less than 0.05.

## Results

### Patient characteristics by weight loss

A total of 2,174 patients were included in this study after excluding patients receiving neoadjuvant or adjuvant therapy, with a history of other cancer, or without weight loss data. All the patients were divided into two groups, no weight loss (-) and weight loss(+), according to whether there was weight loss before surgery. Patient characteristics were shown in Table 1, which indicated that weight loss (+) was significantly associated with age (*P*=0.001), alcoholism (*P*<0.001), tumor location (*P*=0.003), pT category (*P*=0.003), and pN category (*P*=0.001).

### Univariate Cox analysis

Univariate Cox analysis showed that group weight loss (+) had a significantly poorer DFS (Mean: 63.3 m vs 76.8 m, *P*<0.001, Table 2) and OS (67.4 m vs 83.3 m, *P*<0.001, Table 2) than group no weight loss (-). Moreover, as shown in Table 2, the male gender, smoking history, alcohol consumption, poor pathological stage, and poor histological differentiation were significantly associated with shorter OS and DFS (*P*<0.05).

### Multivariate survival analysis

**Figure 1:**
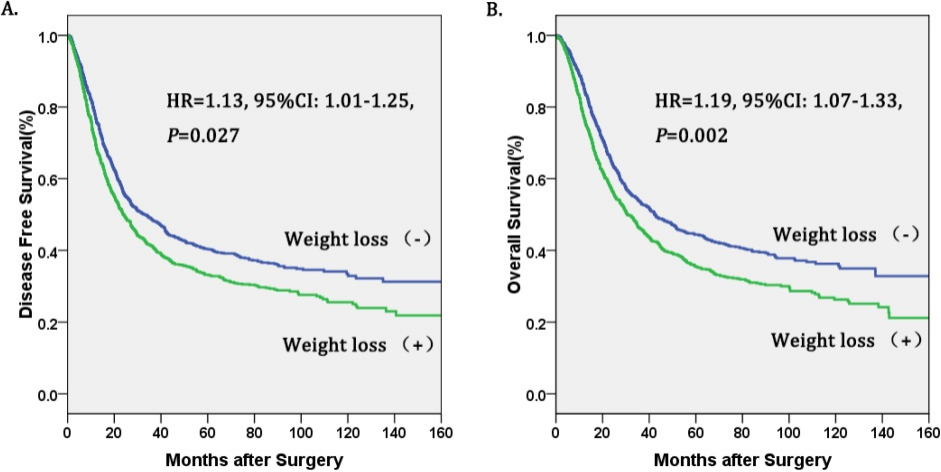
** Kaplan-Meier curves of A.** Disease free survival (DFS) subdivided by preoperative weight loss in patients with esophageal cancer, **B.** Overall survival (OS) subdivided by preoperative weight loss in patients with esophageal cancer.

The Cox proportional hazards regression indicated that weight loss (+) was an independent prognostic factor in operable esophageal cancer (Table 3, figure 1). In the final multivariate survival analysis with adjustment for covariates, we found that patients in the weight loss (+) group had a 19% higher risk of death (HR=1.19, 95%CI: 1.07-1.33, *P*=0.002) and a 13% higher risk of disease progression (HR=1.13, 95%CI: 1.01-1.25, *P*=0.027) than patients in the no weight loss (-) group. Besides, alcohol consumption^17^ and pathological stage were also independent prognostic factors for operable esophageal cancer.

### Subgroup analysis

**Figure 2:**
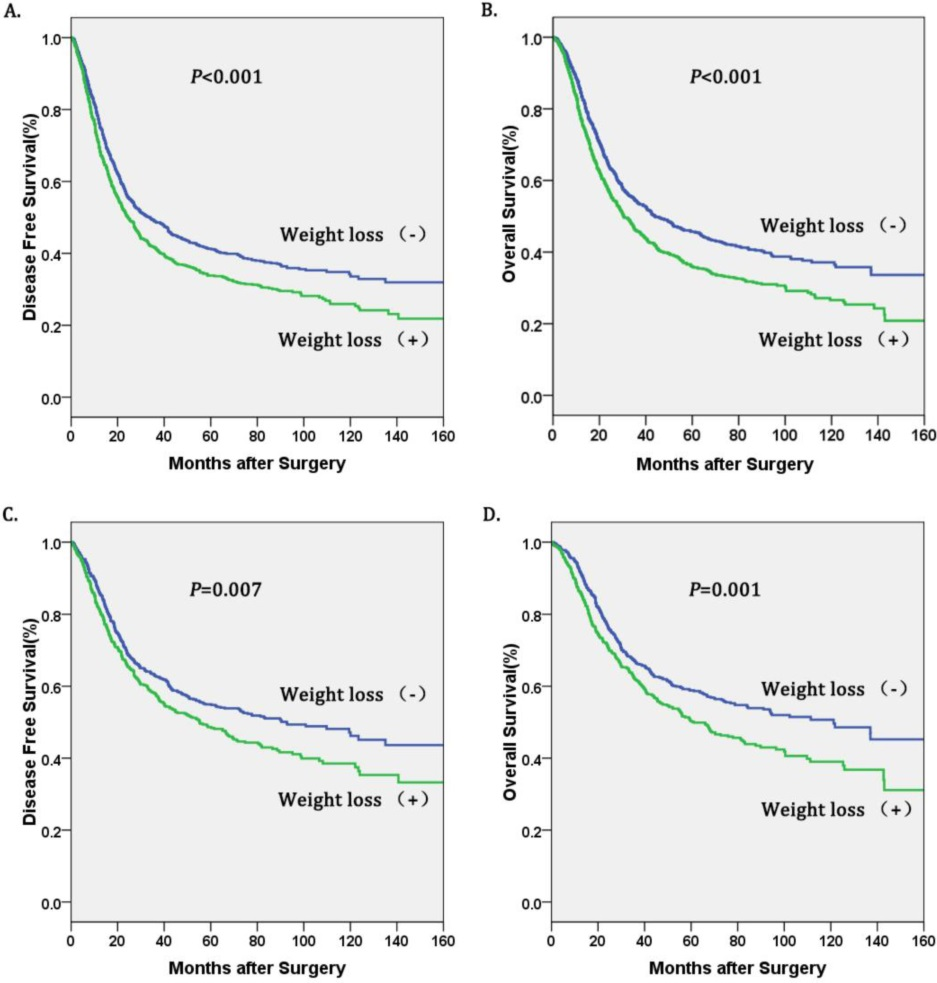
** Kaplan-Meier curves of A.** Disease free survival (DFS) subdivided by preoperative weight loss in patients with esophageal squamous cell carcinoma, **B.** Overall survival (OS) subdivided by preoperative weight loss in patients with esophageal squamous cell carcinoma, **C.** Disease free survival (DFS) subdivided preoperative weight loss in esophageal cancer patients with pathological stage I-II, **D.** Overall survival (OS) subdivided by preoperative weight loss in in esophageal cancer patients with pathological stage I-II.

Univariate survival analyses stratified by histology and TNM stage were performed. We found that weight loss (+) was associated with decreased OS and DFS in patients with esophageal squamous cell carcinoma (ESCC), and in TNM stage I-II (Table 4, figure 2). However, there was no significant association between weight loss (+) and DFS or OS in patients with TNM stage III-IV (Table 4, Figure 2).

## Discussion

In our study, preoperative weight loss was related to age, alcohol consumption, tumor location, pT category, pN category and pathological stage. Preoperative weight loss was associated with shorter OS and DFS, which meant poorer prognosis in patients who underwent surgery, and we also shown that preoperative weight loss was a prognostic factor for postoperative prognosis. In subgroup analysis, we found that weight loss (+) was associated with shorter OS and DFS in patients with esophageal squamous cell carcinoma (ESCC), as well as TNM stage (I-II).

Weight loss is a comment symptom of esophageal cancer that approximately 57% to 85% patients experience before surgery 3,5. It has been reported that preoperative weight loss is a prognostic factor in colon 7, stomach 8, cervical 9 and lung cancers 10. In our study, esophageal cancer patients who suffered preoperative weight loss had decreased OS and DFS compared with those without weight loss. More importantly, multivariate analysis demonstrated that preoperative weight loss was an independent prognostic factor in esophageal cancer. Patients with preoperative weight loss had a 19% higher risk of death (HR=1.19, 95%CI: 1.07-1.33, *P*=0.002) and a 13% higher risk of disease progression than those without weight loss. The previous studies also showed that preoperative weight loss was significantly associated with poor prognosis 11-13, which is consistent with our study.

In addition, multivariate analysis in our study indicated that preoperative weight loss is an independent prognostic factor of esophageal cancer. According to previous research, dysphagia, decreased oral intake, systemic inflammation, and mental factors were likely to lead to systemic inflammation and cachexia presented as preoperative weight loss 4-6. Preoperative weight loss may mean poorer nutritional status and less tolerance for surgery, which implies a worse prognosis. Interestingly, some inflammatory markers and nutritional markers such as blood neutrophils and serum fibrinogen level have also been reported as prognostic risk factors for esophageal cancer which confirm our result further 18,19. Therefore, preoperative weight loss in esophageal cancer may serve as a marker of more severe systemic inflammation, cachexia, malnutrition and worse prognosis 20. Our study suggest that preoperative correction of nutritional status and reduction of inflammatory response in patients with esophageal cancer may be helpful to the prognosis of patients with esophageal cancer, but further studies are needed to prove this. Finally, further subgroup analysis showed that preoperative weight loss was significantly associated with shorter OS and DFS in patients with esophageal squamous cell carcinoma (ESCC) and early pathological stage (I-II), but not associated with patients with esophageal adenocarcinoma, and pathological stage (III-IV).

One advantage of our study is the large cohort of patients after transthoracic esophagectomy with radical lymphadenectomy in China. Previous studies have shown that preoperative weight loss predicted a worse prognosis, the largest sample size included 922 patients. Our study included 2174 patients that made our results more reliable. Additionally, our study has collected nearly complete survival data from patients after surgery. However, there are certain limitations in this study. First of all, it was a retrospective study focusing on the Asian population, which could lead to selection bias. Second, we used self-reported instead of measured weight and information to determine preoperative weight loss, which might result in imprecise information. Finally, our study simply stratified patients into two groups according to whether they had preoperative weight loss, which leads to analysis gaps and increases the impact of confounders. In the future, multi-center studies and prospective clinical studies as well as laboratory researches are necessary to determine the prognostic role of preoperative weight loss in esophageal cancer.

## Conclusions

In conclusion, our study with a large cohort of 2174 Chinese patients who had undergone surgery provided more definite and quantitative evidence for the association between preoperative weight loss and shorter OS and DFS. More importantly, preoperative weight loss is a factor for postoperative prognosis in esophageal cancer.

## Figures and Tables

**Table 1:** clinicopathologic characteristics in 2174 patients with esophageal cancer

Prognostic factor	Patients (%) N=2174	Weight loss (-)(%) N=1155	Weight loss (+)(%) N=1019	*P*
**Hp**				0.300
ESCC	1894(87.1)	1018(88.1)	876(86.0)	
EA	195(9.0)	94(8.1)	101(9.9)	
Others	85(3.9)	43(3.8)	42(4.1)	
**Age**				**0.001**
≤58 years	1311(60.3)	658(57.0)	653(64.1)	
>58years	863(39.7)	497(43.0)	366(35.9)	
**Gender**				0.996
Females	497(22.9)	264(22.9)	233(22.9)	
Males	1677(77.1)	891(77.1)	786(77.1)	
**Smoking**				0.203
Never	777(35.7)	427(37.0)	350(34.3)	
Ever (former + current)	1397(64.3)	728(63.0)	669(65.7)	
**Alcohol**				**<0.001**
Never	1491(68.6)	833(72.1)	658(64.6)	
Ever (former + current)	683(31.4)	322(27.9)	361(35.4)	
**Radicality of surgery**				0.788
R0	2004(92.2)	1063(92.0)	941(92.3)	
R1	170(7.8)	92(8.0)	78(7.7)	
**Differentiation**				0.780
G1	1472(67.7)	779(67.4)	693(68.0)	
G2-3	702(32.3)	376(32.6)	326(32.0)	
**Tumor location**				**0.003**
Upper	391(18.0)	229(19.8)	162(15.9)	
Middle	1136(52.3)	619(53.6)	517(50.7)	
Lower	452(20.8)	213(18.4)	239(23.5)	
EGJ	195(8.9)	94(8.2)	101(9.9)	
**pT category**				**0.003**
T1-2	662(30.6)	383(33.2)	279(27.4)	
T3-4	1512(69.4)	772(66.8)	740(72.6)	
**pN category**				**0.001**
N0	1109(51.0)	628(54.4)	481(47.2)	
N1-3	1065(49.0)	527(45.6)	538(52.8)	

Hp, histopathology; ESCC, esophageal squamous cell carcinoma; EA, esophageal adenocarcinoma; EGJ, esophagogastric junction; G, grade. Bold values are statistically significant (*P* < 0.05).

**Table 2:** Univariate survival analysis for overall survival and disease free survival in patients with esophageal cancer

Prognostic factor	Disease-free survival (Months)				Overall survival (Months)		
	Mean	Median	*P*		Mean	Median	*P*
**Hp**			0.252				0.133
ESCC	71.7	27.8			77.0	36.7	
EA	53.2	23.8			57.2	31.2	
Others	62.9	32.0			70.4	40.3	
**Age**			0.335				**0.028**
≤58 years	73.0	27.0			79.5	37.9	
>58 years	66.4	27.8			70.2	35.0	
**Gender**			**<0.001**				**<0.001**
Females	82.2	39.2			89.2	54.8	
Males	66.2	25.4			34.1	54.8	
**Smoking**			**<0.001**				**<0.001**
Never	81.3	36.3			86.8	46.7	
Ever (former + current)	63.8	24.1			69.3	31.7	
**Alcohol**			**<0.001**				**<0.001**
Never	76.9	33.1			82.8	43.5	
Ever (former + current)	55.7	20.3			60.0	25.3	
**Weight loss**			**<0.001**				**<0.001**
Weight loss (-)	76.8	32.4			83.3	42.4	
Weight loss (+)	63.3	24.1			67.4	30.8	
**Differentiation**			**<0.001**				**<0.001**
G1	77.6	34.4			82.6	43.4	
G2-3	55.6	21.3			61.4	26.8	
**Tumor location**			0.345				0.164
Upper	62.5	29.6			66.0	39.8	
Middle	72.9	28.3			78.8	38.7	
Lower	67.5	25.0			72.9	32.7	
EGJ	43.1	24.8			46.8	34.1	
**Pathological stage**			**<0.001**				**<0.001**
Stage I-II	95.5	71.6			100.1	84.0	
Stage III-IV	39.7	15.8			45.7	20.8	

EGJ, esophagogastric junction; G, grade; HR, hazard ratio; 95% CI, 95% confidence interval. Bold values are statistically significant (*P* < 0.05).

**Table 3:** Multivariate survival analysis for overall survival and disease free survival in patients with esophageal cancer

Prognostic factor	Disease-free survival			Overall survival	
	HR(95%CI)	*P-*value		HR(95%CI)	*P-*value
**Age**	-	*-*		1.19(1.06-1.33)	**0.002**
**Gender**	0.99(0.83-1.18)	0.879		0.94(0.79-1.13)	0.519
**Smoking**	1.09(0.94-1.28)	0.265		1.10(0.97-1.25)	0.130
**Alcohol**	1.33(1.19-1.49)	**<0.001**		1.36(1.22-1.53)	**<0.001**
**Weight loss**	1.13(1.01-1.25)	**0.027**		1.19(1.07-1.33)	**0.002**
**Differentiation**	1.27(1.13-1.42)	**<0.001**		1.27(1.13-1.42)	**<0.001**
**Pathological stage**	2.44(2.18-2.72)	**<0.001**		2.42(2.16-2.72)	**<0.001**

HR, hazard ratio; 95% CI, 95% confidence interval. Bold values are statistically significant (*P* < 0.05).

**Table 4:** Subgroup analysis by weight loss for overall survival and disease free survival in patients with esophageal cancer

Prognostic factor	Disease free Survival (Months)				Overall Survival (Months)		
	Mean	Median	*P*		Mean	Median	*P*
**Hp**							
ESCC			**<0.001**				**<0.001**
Weight loss (-)	77.9	33.4			84.5	43.9	
Weight loss (+)	64.0	24.7			67.9	30.5	
EA			0.559				0.738
Weight loss (-)	43.6	24.6			46.9	30.2	
Weight loss (+)	52.4	21.3			56.9	34.3	
Others			0.103				0.143
Weight loss (-)	51.8	39.3			56.1	42.1	
Weight loss (+)	51.0	22.0			60.4	32.5	
**TNM stage**							
Stage I-II			**0.007**				**0.001**
Weight loss (-)	100.5	92.7			106.4	121.2	
Weight loss (+)	87.6	55.4			90.2	61.2	
Stage III-IV			0.339				0.198
Weight loss (-)	41.1	16.8			47.3	21.9	
Weight loss (+)	38.5	15.2			44.0	19.4	

Hp, histopathology; ESCC, esophageal squamous cell carcinoma; EA, esophageal adenocarcinoma. Bold values are statistically significant (*P* < 0.05).

## References

[1] Chen W, Zheng R, Baade PD, Zhang S, Zeng H, Bray F, Jemal A, Yu XQ, He J (2016). Cancer statistics in China, 2015. CA: A Cancer Journal for Clinicians.

[2] Shapiro J, van Lanschot J, Hulshof M, van Hagen P, van Berge HM, Wijnhoven B, van Laarhoven H, Nieuwenhuijzen G, Hospers G, Bonenkamp JJ, Cuesta MA, Blaisse R, Busch O, Ten KF, Creemers GM, Punt C (2015). Neoadjuvant chemoradiotherapy plus surgery versus surgery alone for oesophageal or junctional cancer (CROSS): long-term results of a randomised controlled trial. LANCET ONCOL.

[3] Kuzu MA, Terzioglu H, Genc V, Erkek AB, Ozban M, Sonyurek P, Elhan AH, Torun N (2006). Preoperative nutritional risk assessment in predicting postoperative outcome in patients undergoing major surgery. WORLD J SURG.

[4] Deans DA, Tan BH, Wigmore SJ, Ross JA, de Beaux AC, Paterson-Brown S, Fearon KC (2009). The influence of systemic inflammation, dietary intake and stage of disease on rate of weight loss in patients with gastro-oesophageal cancer. Br J Cancer.

[5] Daly JM, Fry WA, Little AG, Winchester DP, McKee RF, Stewart AK, Fremgen AM (2000). Esophageal cancer: results of an American College of Surgeons Patient Care Evaluation Study. J Am Coll Surg.

[6] Anandavadivelan P, Lagergren P (2016). Cachexia in patients with oesophageal cancer. NAT REV CLIN ONCOL.

[7] Lavin P, Mittelman A, Douglass HJ, Engstrom P, Klaassen D (1980). Survival and response to chemotherapy for advanced colorectal adenocarcinoma: an Eastern Cooperative Oncology Group report. CANCER-AM CANCER SOC.

[8] Haugstvedt TK, Viste A, Eide GE, Soreide O (1991). Factors related to and consequences of weight loss in patients with stomach cancer. The Norwegian Multicenter experience. Norwegian Stomach Cancer Trial. CANCER-AM CANCER SOC.

[9] Kastritis E, Bamias A, Bozas G, Koutsoukou V, Voulgaris Z, Vlahos G, Rodolakis A, Gika D, Papadimitriou C, Dimopoulos MA (2007). The impact of age in the outcome of patients with advanced or recurrent cervical cancer after platinum-based chemotherapy. GYNECOL ONCOL.

[10] Jeremic B, Shibamoto Y (1995). Pre-treatment prognostic factors in patients with stage III non-small cell lung cancer treated with hyperfractionated radiation therapy with or without concurrent chemotherapy. LUNG CANCER.

[11] Yu X, Yang J, Chen T, Liu Y, Xue W, Wang M, Bai S (2018). Excessive Pretreatment Weight Loss Is a Risk Factor for the Survival Outcome of Esophageal Carcinoma Patients Undergoing Radical Surgery and Postoperative Adjuvant Chemotherapy. CAN J GASTROENTEROL.

[12] Hynes O, Anandavadivelan P, Gossage J, Johar AM, Lagergren J, Lagergren P (2017). The impact of pre- and post-operative weight loss and body mass index on prognosis in patients with oesophageal cancer. European Journal of Surgical Oncology (EJSO).

[13] van der Schaaf MK, Tilanus HW, van Lanschot JJB, Johar AM, Lagergren P, Lagergren J, Wijnhoven BPL (2014). The influence of preoperative weight loss on the postoperative course after esophageal cancer resection. The Journal of Thoracic and Cardiovascular Surgery.

[14] Skipworth J, Foster J, Raptis D, Hughes F (2009). The effect of preoperative weight loss and body mass index on postoperative outcome in patients with esophagogastric carcinoma. DIS ESOPHAGUS.

[15] Zou J, Chen J, Xie X, Liu Z, Cai X, Liu Q, Wen J, Zhang S (2019). Hepatitis B Virus Infection is a Prognostic Biomarker for Better Survival in Operable Esophageal Cancer: Analysis of 2,004 Patients from an Endemic Area in China. Cancer Epidemiol Biomarkers Prev.

[16] Huang Q, Luo K, Chen C, Wang G, Jin J, Kong M, Li B, Liu Q, Li J, Rong T, Chen H, Zhang L, Chen Y, Zhu C, Zheng B, Wen J (2016). Identification and Validation of Lymphovascular Invasion as a Prognostic and Staging Factor in Node-Negative Esophageal Squamous Cell Carcinoma. J THORAC ONCOL.

[17] Huang Q, Luo K, Yang H, Wen J, Zhang S, Li J, Ela BA, Liu Q, Yang F, Zheng Y, Hu R, Chen J, Fu J (2014). Impact of alcohol consumption on survival in patients with esophageal carcinoma: a large cohort with long-term follow-up. CANCER SCI.

[18] Zhang S, Lei Y, Cai X, Yang H, Xia X, Luo KJ, Su C, Zou J, Zeng B, Hu Y, Luo H (2016). Preoperative serum fibrinogen is an independent prognostic factor in operable esophageal cancer. Oncotarget.

[19] Sharaiha RZ, Halazun KJ, Mirza F, Port JL, Lee PC, Neugut AI, Altorki NK, Abrams JA (2011). Elevated preoperative neutrophil:lymphocyte ratio as a predictor of postoperative disease recurrence in esophageal cancer. ANN SURG ONCOL.

[20] Shen S, Araujo JL, Altorki NK, Sonett JR, Rodriguez A, Sungur-Stasik K, Spinelli CF, Neugut AI, Abrams JA (2017). Variation by stage in the effects of prediagnosis weight loss on mortality in a prospective cohort of esophageal cancer patients. DIS ESOPHAGUS.

